# Behavioral Problems in Fragile X Syndrome: A Review of Clinical Management

**DOI:** 10.7759/cureus.21840

**Published:** 2022-02-02

**Authors:** Michael Davidson, Sneha A Sebastian, Yoanna Benitez, Shreeya Desai, Jonathan Quinonez, Samir Ruxmohan, Joel D Stein, Wilson Cueva

**Affiliations:** 1 Pediatrics, Larkin Community Hospital, South Miami, USA; 2 Research, Larkin Community Hospital, South Miami, USA; 3 Neurology/Osteopathic Neuromuscular Medicine, Larkin Community Hospital, South Miami, USA; 4 Neurology, Larkin Community Hospital, South Miami, USA; 5 Osteopathic Neuromuscular Medicine, Family Medicine, Sports Medicine, Pain Medicine, Lake Erie College of Osteopathic Medicine (LECOM) Bradenton, Bradenton, USA; 6 Pain Management, Osteopathic Neuromuscular Medicine, Sports Medicine, Larkin Community Hospital, South Miami, USA

**Keywords:** psychopharmacology, fmr1, clinical management, behavioral problems, behavioral treatment, fragile x syndrome

## Abstract

Fragile X syndrome (FXS) is noted to be the leading cause of inherited intellectual disabilities and is caused by expansive cytosine-guanine-guanine (CGG) trinucleotide repeats in the fragile X mental retardation 1 gene (FMR1). FXS can display a wide range of behavioral problems in addition to intellectual and developmental issues. Management of these problems includes both pharmacological and non-pharmacological options and research on these different management styles has been extensive in recent years. This narrative review aimed to collate recent evidence on the various management options of behavioral problems in FXS, including the pharmacological and non-pharmacological treatments, and also to provide a review of the newer avenues in the FXS treatment.

## Introduction and background

Fragile X syndrome (FXS) is an X-linked dominant disorder noted to be the leading cause of inherited intellectual disability affecting approximately 1/4,000 males [1]. FXS is caused by cytosine-guanine-guanine (CGG) trinucleotide repeats in the fragile X mental retardation 1 (FMR1) gene located in chromosome Xq27.3 [1]. Expansions of more than 200 repeats are known as the full mutation and patients with at least 200 repeats are noted to have FXS. FXS causes a variety of clinical and behavioral abnormalities, which consequently affect physical and mental health, including learning and behavior. Presently, there are no specific management strategies to completely cure individuals with FXS; however, improvements in the quality of life of these patients can be achieved by providing treatments for challenging behavioral problems. The need to compare both pharmacological and non-pharmacological treatments for behavioral problems in FXS is essential to provide the most up-to-date treatment strategies. This paper will serve as a literature review of both the pharmacological and non-pharmacological management of behavioral concerns of FXS and provide a review of treatments available for managing behavioral problems in patients with FXS.

## Review

We did a literature search of relevant articles from the databases, namely, PubMed and Google Scholar, until January 7, 2022, using the following keywords: "fmr1," "clinical management," "behavioral problems," "behavioral treatment," "fragile X syndrome," and "psychopharmacology." Articles had to meet four inclusion criteria: (1) studies that included diagnosed cases of FXS; (2) general studies conducted in FXS patients to assess the behavioral problems and their management; (3) all study designs, including clinical trials, observational studies, systematic and literature reviews, and case reports that assessed the clinical management of behavioral problems in FXS; and (4) studies published in the last two decades and written in the English language. We excluded articles published in other languages.

Epidemiology

Estimates for the prevalence of FXS vary considerably. However, a recent meta-analysis has shown the prevalence of the full mutation was 1.4 per 10,000 males and 0.9 per 10,000 females; 1:7,143 and 1:11,111, respectively, within the total population [2]. The higher rate of males is explained by the X-linked nature of the disorder; however, a number of females are still impacted by the disorder. Concerning behavioral aspects of the disorder, males with the full mutation show features of autism spectrum disorder (ASD) in about 90% of cases with 60% meeting diagnostic criteria for ASD [3]. Female prevalence of comorbid ASD is noted to be 17% [3]. Additionally, some studies have demonstrated an inverse relationship between fragile X mental retardation protein (FMRP) expression and the likelihood of receiving a diagnosis of ASD in males with FXS [3]. Many of the behavioral features of ASD are also displayed in patients with FXS, even if the diagnosis of ASD is not fully met, these include poor eye contact, difficulty with relationships, the performance of repetitive tasks, and distress of small changes in daily activities. Patients with FXS may also demonstrate self-injurious behaviors (SIB) secondary to this repetition as well as a way to alleviate stress from external stimuli [2,3]. In addition to the features of ASD, patients with FXS may also demonstrate clinical features of attention deficit hyperactivity disorder (ADHD), including inattentiveness, impulsivity, and restlessness. Meta-analysis suggests that up to 80% of patients with FXS meet the diagnostic criteria for ADHD [4].

Clinical features of fragile X syndrome

Clinical features of FXS vary greatly depending on the number of CGG repeats [1]. The number of repeats may be considered normal, generate the premutation, or be the full mutation associated with the disorder. Consequently, the amount of FMRP produced is dependent on the number of repeats, with more repetitions producing less FMRP and resulting in a more severe condition [1,2,5]. Symptoms are not exhibited by individuals with the normal allele (5-44 repeats) and premutation allele (55-200 repeats) and are therefore not diagnosed with FXS. Individuals with the full mutation allele (>200) are diagnosed with FXS and experience a variety of symptoms and clinical features [5]. These clinical features can be subclassified into physical, psychological/psychiatric, and developmental issues. Physical symptoms include a long and narrow face, prominent ears and jaw, microcephaly, and macroorchidism (in males) post-puberty. Patients with FXS may also suffer from a number of comorbid medical issues, including strabismus, hearing loss as a consequence of recurrent ear infections, obesity, joint hypermobility, and seizures [6]. In addition to physical conditions and comorbid medical issues, children with FXS also display a wide range of psychological and behavioral symptoms. These include ADHD, ASD, aggressiveness, and anxiety, as well as delays in development, both early on and throughout life [3,7]. For example, delays in language development are often seen in patients with full mutation. Additionally, patients with FXS have varying degrees of intellectual disabilities. The severity of the intellectual disability is dependent on the level of methylation of the repeated segment with more methylation being associated with more profound intellectual deficits and lower adult intelligence quotient (IQ) levels [8,9]. The average IQ of males with the full mutation has been shown to be about 40 [8]. In contrast, patients with more mild intellectual defects have lower levels of methylation [1]. Patients may also display deficiencies in visuospatial as well as mathematical abilities and show difficulty in social settings, symptoms often associated with ASD [10]. Clinical features of FXS can also be categorized based on prevalence by gender with higher prevalence seen in males compared to females.

Behavioral challenges in FXS

FXS is often associated with several psychopathological conditions, including anxiety and depression. These features often occur in both childhood and adulthood and can cause significant impairment in daily functioning [3,7,11,12]. In addition to these issues, individuals with FXS often present with behavioral abnormalities resulting in learning disabilities and social problems. These features can be challenging to their families and peers and could hinder their interactions within the society. The most common behavioral problems seen in FXS include behaviors associated with ADHD, which include hyperactivity and restlessness. In severe cases, patients may display aggression and behaviors causing self-injury. Patients with FXS may also suffer from generalized anxiety disorder and problems with social interaction, including social anxiety and withdrawal (Table 1).

**Table 1:** Common behavioral problems in FXS ADHD, attention deficit hyperactivity disorder; FXS, fragile X syndrome; DSM-V, Diagnostic and Statistical Manual of Mental Disorders, 5th Edition; ASD, autism spectrum disorder.

Behavioral problems	Symptoms
Anxiety disorders	Anxiety and social withdrawal are considered core features of FXS. Certain individuals show social problems with social interaction and communication. These individuals also showed other DSM-V anxiety disorders like separation anxiety, social phobia, agoraphobia, selective mutism, and obsessive-compulsive disorder. Individuals with FXS are also prone to develop depression along with anxiety disorders [10].
ADHD	Impaired ability to maintain attention, difficulty focusing on specific tasks, and hyperactive behavior such as fidgeting or impulsive actions. About one-third of FXS individuals can have ASD combined with hyperactive symptoms, which can affect communication and social interaction. Features of autism in FXS individuals include perseverative speech and behavior, poor eye contact, and social anxiety [4].
Aggression/self-injurious behavior	Overactivity and impulsivity seen in FXS individuals are the main factors contributing to aggression and self-injurious behavior. Hand biting is the most common self-injurious behavior. Hitting and kicking are the most frequent forms of aggressive behaviors [11].

Management strategies for the behavioral problems in FXS

The management of psychological/behavioral issues associated with FXS is achieved through both pharmacological and non-pharmacological techniques. Due to the high prevalence of ASD and ADHD in those with the full mutation, therapies used to treat behavioral aspects of ASD, and ADHD may be beneficial in those with FXS [3,4]. Additionally, many of the psychological and behavioral issues seen in those with ASD and ADHD are also present in patients with FXS [3,4]. Understanding the pathogenesis of the disorder allows for the application of pharmacological management, which targets specific cellular pathways affected by FXS. This knowledge has already been used to develop possible pharmacological treatments. The use of both pharmacological and non-pharmacological treatments for the management of behavioral issues associated with FXS has been studied extensively and the comparison between the two is essential to determine the most appropriate course of action for the treatment of behavioral problems. Following are the non-pharmacological, pharmacological, and newer advancements in the clinical management of behavioral challenges in FXS.

Non-pharmacological management

The application of non-pharmacological therapies used in patients with ASD and ADHD has been shown to be beneficial in the management of similar concerns in FXS. These therapies have been shown to improve behavioral issues such as anxiety, aggression, and SIB. The application of these treatment methods may prove beneficial in patients with FXS. However, the use of behavioral services, such as applied behavioral analysis, social training, tutoring, and vocational training, in patients of FXS who meet diagnostic criteria of ASD is of concern considering the core nature of ASD and its associated cognitive and learning challenges [13]. The application of some of these therapies has shown to be beneficial in addressing some of the problem behaviors of FXS, which will be further detailed throughout this paper.

In regards to anxiety, patients with ASD have shown improvements with the use of cognitive-behavioral therapy (CBT). Due to the high number of patients with FXS who also meet diagnostic criteria for ASD, the use of similar therapies may prove to be beneficial and should be explored further. Specifically, van Steensel and Bögels demonstrated the effectiveness of CBT to manage anxiety symptoms in patients with ASD [14]. They further demonstrated no significant difference between patients with ASD and those without it and the effectiveness of CBT in the management of anxiety, reporting that 61% of patients with ASD were free of their anxiety disorder vs. 64% of patients without ASD [14]. The application of this study to children with FXS may be limited since the majority of children had high functioning ASD and its effectiveness in patients with more severe ASD, as well as FXS, specifically, should be implemented.

Patients with FXS often exhibit aggressive behavior. The use of non-pharmacological therapies may prove beneficial in dealing with this aggression and has previously demonstrated effectiveness in patients with ASD. In addition to aggression, irritability and SIB have shown improvement through the use of these therapies in patients with ASD and ADHD, and studies in patients with FXS have been promising. The American Academy of Pediatrics recommends the use of function-based, patient-specific treatment programs tailored to specific situations in which SIB occurs. However, the application of these therapies for ASD may be limited in patients with FXS, and proper training of therapists to distinguish differences between the disorders is essential [15]. One of the most promising therapies in managing SIB in patients with FXS is functional communication training (FCT), which has shown marked improvement in SIB and aggression in patients with FXS [2,7,16]. Additionally, Hall et al. demonstrated its usefulness as a telehealth method in patients with FXS and noted similar reductions of Aberrant Behavior Checklist-Community (ABC-C) scale aggression scores compared with patients treated in office settings. The results of the study may prove beneficial in patients with FXS who are unable to meet with therapists in their areas trained in FXS and may provide increased improvement of the disease [17]. These findings further suggest the use of non-pharmacological management in dealing with aggression and SIB in patients with FXS and allow for better treatment options. The ability to utilize telehealth methods also allows for better availability for patients and helps to provide therapists better trained in dealing with FXS.

Pharmacological management

The pathogenesis of FXS allows for use of pharmacological management that targets pathways impacted by FXS. A number of drugs have been studied and a review of these treatment methods is required to better manage behavioral issues of FXS.

Selective Serotonin Reuptake Inhibitors

The pharmacological management of anxiety in patients with FXS is often achieved through the use of selective serotonin reuptake inhibitors (SSRIs), which inhibit the reuptake of serotonin [11]. Studies in individuals with ASD have shown decreased metabolism of tryptophan to serotonin [18]. Due to the high comorbidity between ASD and FXS, the pharmacological options used to manage anxiety in ASD have been applied to patients with FXS. Sertraline is the most commonly used SSRI to treat anxiety in patients with FXS [11]. Its use was previously approved by the Food and Drug Administration (FDA) in children (6-17 years old) for the treatment of the obsessive-compulsive disorder (OCD) [11,19]. The use of sertraline in patients with FXS showed improvement in anxiety, hyperarousal, and language deficiencies. A retrospective study by Winarni et al. compared children (aged 18 months to 6 years) with FXS, treated with and without sertraline in dosage of 2.5 mg to 12.5 mg/day for three months, beginning at 18 months of age [18]. The dose of sertraline was increased as tolerated, and side effects such as hyperarousal, irritability, and aggression were noted with higher doses [19]. Mullen Scales of Early Learning (MSEL) showed improvement in expressive and receptive language development in children with FXS treated with sertraline [19]. In addition to the benefit of language development, sertraline at doses between 2.5 mg to 5 mg/day has been shown to cause improvements in anxiety, irritability, and social deficits in children with FXS [14]. Therefore, demonstrating the efficacy of low-dose sertraline (2.5 mg to 5 mg/day) in attaining language milestones and reducing anxiety in children with FXS with minimal adverse effects and drug interactions [11].

Cannabidiol

In addition to SSRIs, the use of cannabidiol (CBD) may prove to be an efficient treatment option for anxiety and other behavioral concerns in patients with FXS [11,20]. CBD exhibits effects on gamma-aminobutyric acid (GABA) and serotonin (5HT1A) receptors and improves the balance between inhibitory and excitatory neurotransmitters [11,21]. The effect on GABAergic neurotransmitters has already demonstrated its effectiveness in treating seizures by increasing the amount of the inhibitory molecule [6,11]. This finding coupled with the fact that seizures are common medical issues in FXS suggests that CBD may be a good targeted therapy for the treatment of FXS. Furthermore, endocannabinoids have been shown to increase excitability in FMR1-knockout (FMR1-KO) mice, further suggesting its use as a targeted treatment for FXS [21].

A number of authors have demonstrated the anxiolytic effects of CBD in generalized anxiety disorder and have further applied this effect to a number of other neurological conditions, including Parkinson's and Huntington's disease [11,20]. Open-label studies in children with FXS in Australia showed improvements in anxiety and depression on the Anxiety, Depression, and Mood Scale (ADAMS) after receiving 250 mg CBD bi-weekly via transdermal patch [11,22,23]. Additional control studies with 200 FXS children demonstrated efficiency in participants with greater than 90% methylation based on the social avoidance subscale of the Aberrant Behavior Checklist-Community for Fragile X Syndrome (ABC-C FXS) scale suggesting CBD can be a promising treatment option for patients with severe clinical features of FXS secondary to increased methylation [22]. In addition to its proposed effectiveness, a number of studies have demonstrated the drug's safety in both human and animal testing [11,22,23]. This suggests the drug is a safe option for use in patients with FXS. Further research is needed to better determine the efficiency of CBD in managing FXS.

Atypical Antipsychotics

One of the most concerning behavioral issues in children with FXS is aggressive and self-injurious behaviors, which may be related to repetitive behaviors displayed by these patients [3,7,16]. Patients who display these behaviors do so with the intent of physical harm but without the intent of death. In those with FXS, SIB showed an incidence of 17-70% and was most commonly reported as self-biting, rubbing, and scratching [16]. Repetitive actions such as limb banging, biting, and hair-pulling may lead to significant harm to the patient, and the need for intervention is essential to ensure the safety of both the patient and caregiver. Examination of both pharmacological and non-pharmacological treatments is essential to prevent SIB. A high portion of FXS patients is treated with antipsychotics for several reasons. The use of risperidone as a pharmacological treatment of SIB associated with FXS has been shown to be beneficial. FDA approval of risperidone in treating aggression and self-injury in children with ASD was achieved in 2006 [16,24]. Placebo-controlled trials have demonstrated the effectiveness of reducing aggression and SIB in patients with ASD, which may be present in patients with FXS. McCracken et al. showed that scores on the Aberrant Behavior Checklist, Irritability (ABC-I) subscale were significantly reduced in the treatment group compared to the placebo at doses of 0.25 mg/day for patients <20 kg and 0.5 mg/day for patients ≥20 kg [24]. Additional placebo-controlled studies also demonstrated similar findings with a significant reduction of the ABC-I subscale score [25]. Dominick et al. demonstrated treatment responsiveness of 33% of participants based on Clinical Global Impressions (CGI) scale score after 22 months of treatment with a mean dose of 2.5 mg/day of risperidone [26]. Further examination of studies shows higher rates of effectiveness of risperidone in managing irritability and SIB in patients with FXS who had comorbid ASD, were older, and had more significant intellectual disabilities [26]. The responsiveness to FXS and ASD may further support its use due to improved outcomes presented by McCracken et al. in the management of aggression and SIB in patients with an ASD.

Riluzole

Riluzole may also be beneficial in the treatment of aggression and SIB in patients with FXS. The drug works by inhibiting glutamate release and enhances glutamate reuptake via inactivated sodium channels [16,27,28]. A large number of clinical trials exploring its usefulness in FXS are currently being conducted [13,27,28]. The use of the medication has shown to be effective at eliminating SIB in patients with severe ASD as well as addressing irritability in ASD and Tourette's syndrome [28]. Patients who were given the medication showed improved behaviors on the Clinical Global Impressions-Improvement (CGI-I) and Clinical Global Impressions-Severity (CGI-S) scales, including a decrease in aggression and SIB [27,28]. Further studies indicated a decrease in repetitive behaviors for these individuals as well. However, clinical studies in patients with FXS showed only minor clinical improvement at similar doses in only 16% of participants [28]. The authors of these studies do note that the shortened timeline of the study may have prevented an appropriate clinical response in the FXS phenotype and suggest longer-term studies [28]. Further studies in children with ASD showed that riluzole may act as an adjunct to risperidone and indicated a greater decrease in the ABC-C aggression scale compared to patients treated with risperidone alone [29]. This combined treatment option should be explored in patients with the FXS phenotype to determine its usefulness in preventing aggression and SIB in those patients.

Metformin

Patients with FXS may display clinical features similar to those with ADHD such as hyperactivity. The management of these features includes both pharmacological and non-pharmacological treatments. Metformin, a drug normally used in the treatment of obesity and diabetes mellitus, is a promising pharmacological treatment for such issues [11,30,31]. An isogenic human stem cell study conducted by Utami et al. in 2020 on elevated de novo protein synthesis in FMRP-deficient human neurons and its correction with metformin showed normalization of protein synthesis in FMRP-deficient human neuronal cells independent of extracellular signal-regulated protein kinases 1 and 2 (ERK1/2) and protein kinase B (Akt) signaling improving behavioral deficits in FXS and normalizing proliferation of FMRP-deficient progenitor human neurons but not neurite outgrowth defects on treatment with metformin, suggesting therapeutic benefit of metformin in improving symptoms [30,31]. Further evidence of metformin's clinical effectiveness was shown in 2018 by Dy et al. [31]. They reported a case series involving individuals of all ages with FXS treated with metformin, showing that doses of 500-1,000 mg twice per day (BID) for adults and 50 mg/day for children aged six months to four years for a period of six months to one year improved hyperactivity, irritability, and social communication [32]. These findings were based on the Aberrant Behavior Checklist, fasting blood sugar, and HbA1c. The authors, therefore, concluded that metformin can be used as a targeted treatment in children and adults with FXS [31]. The authors further noted improvements in language and behavior related to the syndrome [31].

Additionally, Biag et al. demonstrated reduction of irritability, repetitive movements, and improvement of language skills in multiple patients receiving 50-300 mg of metformin daily [32]. Their findings supported previous findings showing a reduction in irritability and hyperactivity in FXS patients receiving metformin. However, Biag et al. also noted increased hyperactivity in one patient receiving metformin and suggested the young age of the patient as a factor; they further recommended that use of metformin to manage FXS should be initiated prior to the age of seven but indicated that some improvement may be delayed in younger patients as the disease progresses [31,32]. Further exploration of metformin’s effectiveness in reducing hyperactivity and irritability is needed. In addition to pharmacological therapy, the use of non-pharmacological methods may be beneficial in managing hyperactivity from FXS.

Minocycline

Minocycline is a tetracycline antibiotic that is used to treat acne. The drug may prove to be useful in patients with FXS due to the inhibition of matrix metalloproteinase-9 (MM-9), which is elevated in FXS secondary to FMRP deficiency [11,12,33]. Animal studies have also demonstrated improved synaptic connections, behaviors, and cognition in FMR1-KO mice that received minocycline; suggesting the drug can be beneficial in FXS [11,33,34]. Open-label studies in patients with FXS have demonstrated improvements in behavioral issues after receiving minocycline [11]. One study showed patients between the ages of 13-32 years with FXS showed improvement in four out of five of the ABC-C subscales, including irritability, stereotypy, hyperactivity, and inappropriate speech [11,35]. Patients also showed improvements in language development as well as decreases in anxiety and showed a significant improvement based on the CGI-I scale [11,35]. Additionally, parents reported improvement in attention and language [11,35]. Adverse effects were most commonly reported as gastrointestinal upset and diarrhea, with one participant reporting worsening hyperactivity [11,36]. In addition to diarrhea, other adverse effects were dizziness and seroconversion to a positive antinuclear antibody (ANA) in two participants [11,36]. Therefore, the use of minocycline requires ANA and liver function studies to ensure the proper safety of the drug [11].

Acamprosate

Acamprosate is currently approved for the maintenance of alcohol abstinence. Due to the drug's effect on GABA and glutamate neurotransmission, the drug may prove to be beneficial in patients with FXS. The drug is thought to exhibit a therapeutic effect via metabotropic glutamate receptor 5 (mGluR5) antagonism [11,12,37,38]. The drug's effectiveness and safety have been demonstrated in clinical trials. A 10-week open-label trial in patients with FXS showed treatment response in 75% of participants who received the drug [11,12,39,40]. The participants demonstrated improvements in social behaviors and hyperactivity [11,12,39,40]. Further studies on the effectiveness of acamprosate for the treatment of behavioral issues in FXS should be further conducted as previous studies have been promising.

Lovastatin

Lovastatin is a 3-hydroxy-3-methylglutaryl coenzyme A (HMG-CoA) reductase inhibitor normally prescribed for the treatment of hyperlipidemia. Studies in FMR1-KO mice showed that the drug prevented epileptogenesis through normalization of protein synthesis, which suggests its usefulness in preventing seizures common among patients with FXS due to increased protein synthesis [11,12,41]. In addition to animal models, a 12-week open-label study demonstrated significant improvement in aberrant behavior in patients with FXS, especially in areas of socialization and communication, and was demonstrated on both the ABC-C and Vineland Adaptive Behavior Scale, Second Edition (VABS-II) [11,12,42]. However, an additional study showed that language improvement in patients who received parent-implemented language intervention (PILI) alone or together with lovastatin was not clinically significantly different but both groups did show a significant improvement compared to baseline [43,44]. This suggests that intensive language therapy can be just as beneficial as pharmacological treatments. Future review of control studies is required to better determine the usefulness of lovastatin in FXS.

New avenues in FXS treatment

New research is on the horizon targeting the neurobiological pathway abnormal in FXS. Pop et al. discuss the need for preclinical research for the development of potential therapeutic agents based on the pathophysiology of FXS rather than the current symptomatic approach for FXS management [45]. According to their review, mGluR5 antagonists could be a great option for the management of FXS as they can modulate mGluR5, which plays an important role in the pathophysiology of FXS by enhanced glutamatergic signaling that leads to increased protein synthesis and defects in synaptic plasticity, including enhanced long-term depression. At present, there is no therapeutic option that could directly reverse the loss of FMRP; however, more research studies about this topic are on the horizon.

## Conclusions

FXS patients can present with several behavioral problems including anxiety, autistic symptoms, and hyperactivity which can be managed by early implementation of developmental and behavioral therapies. Behavioral management tool includes both medication(s) and/or non-pharmacological management options like occupational therapy, speech-language therapy, and combined efforts of a transdisciplinary team. Early introduction of this treatment strategy with an individualized approach can improve the quality of life of FXS patients. Due to the similarities between FXS and ASD, many of the therapeutic techniques for ASD have been applied to FXS. However, the additional diagnosis of ADHD or its symptoms in patients with FXS may further complicate therapies normally used to treat behavioral problems in ASD alone. Patients with FXS may exhibit higher levels of hyperactivity and inattentiveness limiting a patient’s full participation in therapy. Therefore, examination of specific therapies as well as the examination of pharmacological interventions for behaviors associated with ADHD seen in patients with FXS is needed to better improve outcomes.

## Appendices

Abbreviations

ABC-C: Aberrant Behavior Checklist-Community

ADHD: Attention deficit hyperactivity disorder

ASD: Autism spectrum disorder

CBD: Cannabidiol

CBT: Cognitive behavioral therapy

FDA: Food and Drug Administration

FMR1: Fragile X mental retardation 1

FMRP: Fragile X mental retardation protein

FXS: Fragile X syndrome

mGluR5: Metabotropic glutamate receptor 5
